# Fibrillin gene family and its role in plant growth, development, and abiotic stress

**DOI:** 10.3389/fpls.2024.1453974

**Published:** 2024-10-29

**Authors:** Ahmed H. El-Sappah, Jia Li, Kuan Yan, ChaoYang Zhu, Qiulan Huang, Yumin Zhu, Yu Chen, Khaled A. El-Tarabily, Synan F. AbuQamar

**Affiliations:** ^1^ College of Agriculture, Forestry, and Food Engineering, Yibin University, Yibin, Sichuan, China; ^2^ Department of Genetics, Faculty of Agriculture, Zagazig University, Zagazig, Egypt; ^3^ Department of Biology, College of Science, United Arab Emirates University, Al Ain, United Arab Emirates

**Keywords:** drought stress, FBNs, PAPS, photosynthetic tissue, plastoglobules, plastoglobulin

## Abstract

Fibrillins (FBNs), highly conserved plastid lipid-associated proteins (PAPs), play a crucial role in plant physiology. These proteins, encoded by nuclear genes, are prevalent in the plastoglobules (PGs) of chloroplasts. FBNs are indispensable for maintaining plastid stability, promoting plant growth and development, and enhancing stress responses. The conserved PAP domain of FBNs was found across a wide range of photosynthetic organisms, from plants and cyanobacteria. FBN families are classified into 12 distinct groups/clades, with the 12th group uniquely present in algal–fungal symbiosis. This mini review delves into the structural attributes, phylogenetic classification, genomic features, protein–protein interactions, and functional roles of FBNs in plants, with a special focus on their effectiveness in mitigating abiotic stresses, particularly drought stress.

## 1 Introduction

Fibrillins (FBNs) were first identified as thread-like or tube-like structures of varying thicknesses in bell pepper (*Capsicum annuum*) and dog rose (*Rosa rugosa*) plants. The name “fibrillins” is derived from the fibrils, the suborganellar structures of chromoplasts where the proteins were initially discovered (Newman et al., 1989; Deruère et al., 1994; Jiang et al., 2020). Plastoglobules (PGs) in chloroplasts and algal eyespots have also been recognized as reservoirs of FBN proteins (Jiang et al., 2020). Consequently, the FBN protein family is known by several names, including plastoglobulin (PGL), plastid lipid-associated protein (PAP), chromoplast-specific carotenoid-related protein (ChrC), and chloroplastic drought-induced stress protein 34 kDa (CDSP 34) (Jiang et al., 2020).

FBN families are split into 12 groups (FBN1–FBN12) spanning a range of taxa, with FBN12 confined to lower algal fungus mutualistic interactions (Lohscheider and Río Bártulos, 2016; Kim et al., 2018). FBN proteins, found in several photosynthetic organisms such as cyanobacteria and plants, share the PAP domain (**Supplementary Figure S1**; Cunningham et al., 2010; Lohscheider and Río Bártulos, 2016; Sun et al., 2022). In addition to the unique PAP domain, the FBN11 group has a protein kinase C (PKC) domain, suggesting that members of this group may have other roles that need further exploration beyond lipoprotein-related functions (Li et al., 2020). Moreover, the FBN family also has a wide range of isoelectric point (pI) and molecular weights, and they are found in a variety of plastids, including chloroplasts, elaioplasts, chromoplasts, and etioplasts, which may indicate different activities (Kim et al., 2018).

Initially, FBNs were believed to be involved in the formation of fibril structures (Lee et al., 2020). However, several investigations have shown that FBNs also regulate carotenoid accumulation and chromoplast fibril formation (Jiang et al., 2020). Additionally, certain FBNs contain lipocalin domains, suggesting a role in metabolite transport (Singh and McNellis, 2011). *FBN* family genes are crucial for chloroplast formation and the regulation of metabolism in various plants (Li et al., 2020). FBN proteins are also essential for photosynthesis, lipoprotein structure development, and other vital biological processes (Kim et al., 2015).

The *FBN* family is significant in plant response to environmental stress. The expression of *FBN* genes is affected by biotic stresses, such as pathogenic bacteria, fungi, and viruses (Singh and McNellis, 2011), as well as abiotic stresses like drought, high light, wounding, cold, heat, herbicide, and heavy metals (Pruvot et al., 1996; Langenkämper et al., 2001; Jones et al., 2006; Leitner-Dagan et al., 2006; Lee et al., 2007; Simkin et al., 2007; Farinati et al., 2009). Nevertheless, FBNs exhibit complex and inconsistent gene regulatory patterns in response to abiotic stress (Singh and McNellis, 2011).

This mini review provides a comprehensive assessment of the structure, phylogenetic classifications, genomic properties, and functional roles of FBNs in plants, with a focus on their response to abiotic challenges, particularly drought stress.

## 2 Molecular characterization of plant FBN proteins

Within chloroplasts, FBNs are found in the stroma, thylakoids, and PGs. Notably, the distribution of all 14 *Arabidopsis* FBNs within chloroplasts is differential. Proteome analysis of PGs, which are thylakoid-associated monolayer lipoprotein particles and lipid storage sites, revealed that 70% of PG proteins are composed of seven FBNs: FBN1a, FBN1b, FBN2, FBN4, FBN7a, FBN7b, and FBN8 (Ytterberg et al., 2006; Lundquist et al., 2012). FBN3a, FBN3b, FBN6, FBN9, and FBN11 are located in the thylakoids, while FBN10 is believed to reside in either PGs or thylakoids. Recent immunoblotting studies have shown that FBN6 is present in both the thylakoid and envelope membranes (Lee et al., 2020), whereas FBN5 is found in the stroma (Kim et al., 2015). Further experiments are required to verify the localization of other FBNs, including FBN1a, FBN1b, FBN5, FBN6, and FBN11.

FBNs are also present in plastids other than chloroplasts in cereals. For instance, in *Brasscia rapa*, the proteins BrPAP1, BrPAP2, and BrPAP3 correspond to *Arabidopsis* FBN1a, FBN1b, and FBN2, respectively. BrPAP1 is located in the anther elaioplast, BrPAP2 in the petal chromoplast, and BrPAP3 in leaf chloroplasts, as demonstrated by tissue expression and lipid analysis (Kim et al., 2001; Suzuki et al., 2013).

**Figure 1:**
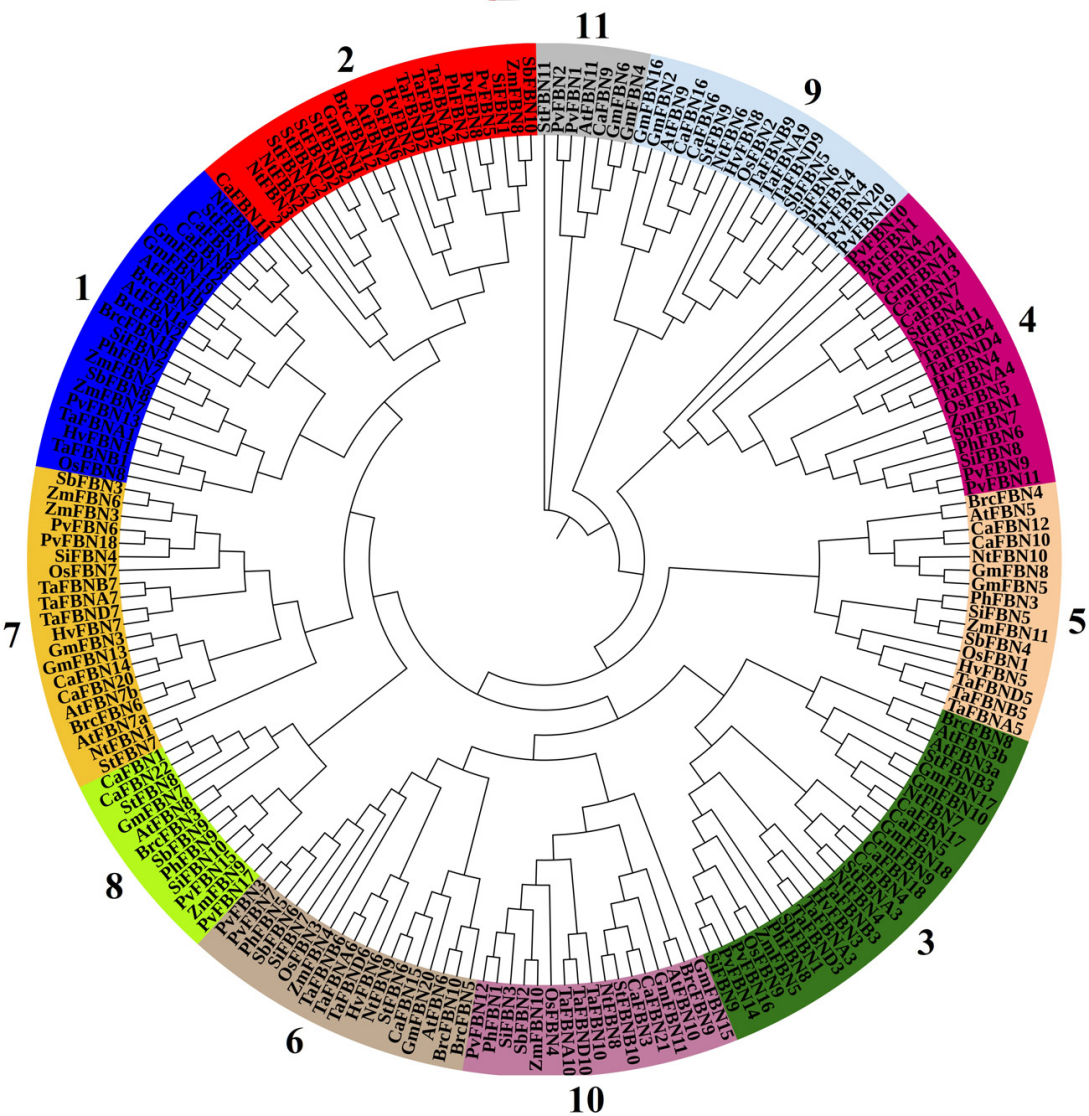
Phylogenetic tree of FBN proteins from various plant species. The 11 plant groups were identified through a phylogenetic analysis of FBN proteins from *Arabidopsis thaliana, Oryza sativa*, *Triticum aestivum*, *Zea mays*, *Sorghum bicolor*, *Solanum tuberosum*, *Panicum hallii*, *Panicum virgatum*, *Setaria italica*, *Hordeum vulgare*, and *Brassica oler*. The phylogenetic tree was constructed using the neighbor-joining method implemented in MEGA7, with 1,000 bootstrap replicates to assess the reliability of the inferred relationships. Bootstrap values are shown at the nodes, and nodes with bootstrap values exceeding 99% are marked with asterisks. Nodes with minimal support (bootstrap values <50%) have been collapsed. FBN, fibrillin.

The FBN protein family comprises 12 subfamilies, including 11 in higher plants and 1 in algae, sharing the PAP domain (PF04755) (**Figure 1**; Lohscheider and Río Bártulos, 2016; Kim et al., 2017). Proteins within these subfamilies have molecular weights ranging between 20 and 42 kDa and pI between 4 and 9. Despite their similar hydrophobic structures, these proteins exhibit diverse biophysical characteristics (Vidi et al., 2006; Lundquist et al., 2012), suggesting specific biological roles for each FBN.

In *Arabidopsis*, FBN proteins feature a conserved hydrophobic domain (lipocalin motif 1) at the N-terminus and specific amino acid residues, including aspartic acid, at the C-terminus (Singh et al., 2010). The three-dimensional structure of FBNs resembles lipocalin, suggesting that the FBN family, which resembles lipocalins, supports the idea that FBNs may possess similar biological activities (Singh et al., 2010; Kim et al., 2015; Lohscheider and Río Bártulos, 2016). In addition, the gene structure and conserved domains of FBN11 differ significantly from those of other FBN members, indicating potential novel functions (Sun et al., 2022).

Recently, FBN gene families have been identified in a wide range of plants (**Supplementary Table S1**). These genes exhibit varying intron numbers, but members of the same group generally share similar intron counts and conserved motif organization, pointing to functional commonality (Sun et al., 2022). However, studies in cucumber (Kim et al., 2018), tomato (Sun et al., 2022), and rice (Li et al., 2020) suggest that members of different groups may perform distinct functions, particularly FBN11.

Collinearity analysis revealed that *FBN* genes in rice and tomato do not share homologous gene pairs, while *Arabidopsis* and tomato share eight homologous gene pairs, suggesting divergence of *FBN* genes between monocots and dicots during evolution (Kim et al., 2018; Sun et al., 2022). The widespread distribution of light and hormone response elements, such as those for ethylene, methyl–jasmonic acid, and abscisic acid (ABA), in the promoter regions of the *FBN* genes in wheat (Jiang et al., 2020) and tomato (Sun et al., 2022) indicates that these genes may play roles in regulating light and hormone pathways.

## 3 Interaction of FBN proteins in plants

FBN proteins are essential for the formation of plastid structures and the regulation of various physiological processes in plants, including stress responses. However, their interactions have not been thoroughly explored (Torres-Romero et al., 2022). The most prevalent proteins found in PGs are FBN1a, FBN1b, FBN2, and FBN4, which together form the core proteome of these structures (Lundquist et al., 2012). Notably, FBN2 exhibits a high PG/stroma abundance ratio of 1,188, indicating that it is predominantly associated with PGs (Lundquist et al., 2012). Despite this, FBN2-GFP suggests that the FBN2 localization suggests that FBN2 may also be evenly distributed across thylakoid membranes or within the stroma (Torres-Romero et al., 2022). The presence of two distinct FBN2 populations might indicate the existence of alternative splice variants or post-translational modifications (Karoulias et al., 2020).

FBN1a has been shown to co-immunoprecipitate with FBN2, and their interaction was confirmed through bimolecular fluorescence complementation (BiFC) in transiently expressed *Nicotiana benthamiana* leaves (Kim and Kim, 2022). In addition, earlier studies demonstrated head-to-tail interactions between FBN1a or FBN1b polypeptides, suggesting their potential to form homodimers, heterodimers, or oligomers *in vivo* (Gámez-Arjona et al., 2014). Under abiotic stress conditions, *fbn* mutants exhibit a significant reduction in anthocyanin production, leading to an upregulation of pigment granules and components of the flavonoid biosynthesis pathway (Youssef et al., 2010). Severe cold and light stress in *fbn1-2* mutants result in necrotic tissues, possibly due to increased lipid peroxidation and reactive oxygen species (ROS) accumulation when photosystem II (PSII) performance declines (Triantaphylidès et al., 2008). FBN2 interacts with allene oxide synthase (AOS), FBN1a, and several other photosynthesis-related proteins, and its disruption may increase PSII susceptibility to damage and impair protein functionality (Torres-Romero et al., 2022). The membrane-associated FBN2 population is more active than its soluble population.

In chloroplasts, FBN5 interacts with solanesyl diphosphate synthases 1 and 2 (SPS1 and SPS2) to generate 45-carbon solanesyl diphosphate, the lipid tail of plastoquinone-9 (PQ-9). PQ-9 acts as a photoprotective antioxidant and is vital for electron transport during photosynthesis (Kim et al., 2015; Havaux, 2020). In addition, FBN4/FIB4 interacts with Fd2, the primary ferredoxin protein in *Arabidopsis* leaves, both *in vitro* and *in vivo* (Wang et al., 2018). Ferredoxins are crucial for delivering electrons to various receptor systems in plastids. The interaction between Fd2 and FBN4 appears to positively regulate *FBN4* expression, as *fd2* knockout mutants show reduced *FBN4* transcript levels, which, in turn, diminishes the innate immunity against *Pseudomonas syringae* pv. tomato (*Pst*) DC3000 (Kim and Kim, 2022).

## 4 Functional characterization of plant *FBN* genes

**Figure 2:**
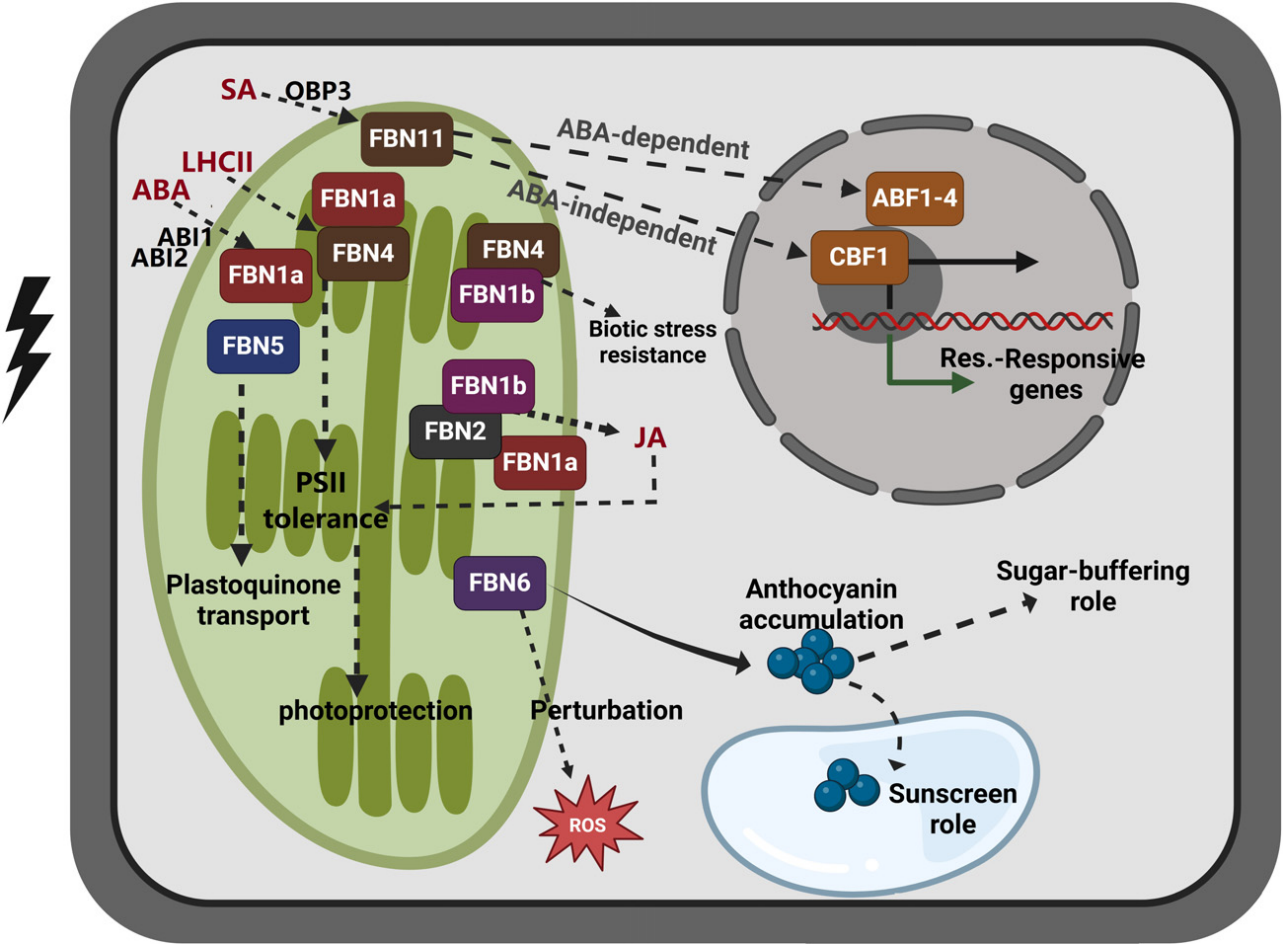
Proposed model of *FBN* family gene function in response to abiotic stress and hormone signaling. FBN1 (FBN1a and FBN1b) and FBN2 are activated by abiotic stress, and they are involved in photoprotection by regulating JA biosynthesis. The expression of *FBN1a*, which is essential for photosynthesis II, has been shown to be influenced by the ABA response regulators ABI1 and ABI2. In proteome analyses of LHCII subcomplexes treated with strong light to induce photoprotective properties, FBN1a and FBN4 were identified. However, the suppression of FBN1b and FBN4 renders plants more susceptible to pathogen infection, suggesting that they may be involved in disease defense. FBN5 interacts with SPS to generate PQ-9, a photoprotective antioxidant. FBN6 facilitates the formation of anthocyanin, which functions as an antioxidant and aids in sugar buffering. FBN11, which is produced by SA treatment and regulated by SA-induced OBP3, may function through either an ABA-dependent process (involving ABF1–4) or an ABA-independent process (involving CBF1). The significance of these *FBN* genes in metabolic control and plant stress responses is underscored by their specialized activities and connections. FBN, fibrillin; SPS, solanesyl diphosphate synthase; SA, salicylic acid; PQ-9, plastoquinone-9; JA, jasmonic acid, ABA, abscisic acid, ABF1–4, ABRE-binding factors; CBF1, CRT/DRE-binding factor 1, also known as *DREB1B*. This figure was made using BioRender.com.

FBNs are distributed across various plastid subcompartments, suggesting distinct roles depending on the type of plastid and lipid. These roles include triacylglycerol, prenyl lipids, carotenoids, and phytohormones such ABA and jasmonic acid (JA) (Singh and McNellis, 2011; Lundquist et al., 2012). Studies using mutant and transgenic plants have explored the roles of *Arabidopsis* FBNs in chloroplast formation and response to light stress (Yang et al., 2006; Singh et al., 2010; Youssef et al., 2010; Kim et al., 2015). However, the precise mechanisms in FBNs’ biological activity remain unclear, as they appear to function through interactions with various proteins or lipids in the plastids. FBNs contribute to multiple plant functions, including PG production, prenylquinone metabolism, pathogen defense, and abiotic stress tolerance (**Table 1**; **Figure 2**).

**Table 1:** Functional attributes of FBNs, highlighting their specific localization within subplastid compartments and their corresponding roles in various cellular processes.

Group	Gene	Plant	Subplastid location	Function	References
Group 1	*AtFBN1a*	*Arabidopsis thaliana*	Plastoglobules	Played a role in PSII photoprotection, starch synthesis, and ABA-mediated photoprotection.	(Yang et al., 2006; Youssef et al., 2010; Lundquist et al., 2012; Gámez-Arjona et al., 2014)
	*GmFBN1a*	*Glycine max*	Plastoglobules	Contributed to plant response to abiotic stress.	(Mutava et al., 2015)
	OsFBN-like protein	*Oryza sativa*	ND	Played a role in cold tolerance.	(Lee et al., 2007)
	*OsFBN1*	*Oryza sativa*	Plastoglobules	Played a role in plastoglobules generation and lipid metabolism.	(Li et al., 2019, 2020)
	TaFBN-like protein	*Triticum aestivum*	ND	Contributed to drought tolerance.	(Cheng et al., 2015)
	*CsaFBN1*	*Cucumis sativus*	Plastoglobules	It demonstrated stimulation and upregulation under intense light and low-temperature stress.	(Kim et al., 2018)
	*SlFBN1*	*Solanum lycopersicum*	Plastoglobules	Played a significant function during tomato fruit differentiation.	(Sun et al., 2022)
	*AtFBN1b*	*Arabidopsis thaliana*	Plastoglobules	Contributed to PSII photoprotection, starch synthesis, and ABA-mediated photoprotection.	(Yang et al., 2006; Youssef et al., 2010; Lundquist et al., 2012; Gámez-Arjona et al., 2014)
	*GmFBN1b*	*Glycine max*	Plastoglobules	Played a role in plant response to abiotic stress.	(Mutava et al., 2015)
Group 2	*AtFBN2*	*Arabidopsis thaliana*	Plastoglobules, stroma, or evenly associated with thylakoid membranes	Regulated JA synthesis under photosynthetic stress and protected PSII from photolysis.	(Youssef et al., 2010; Lundquist et al., 2012)
Group 3	*AtFBN3a*	*Arabidopsis thaliana*	ND	ND	(Lundquist et al., 2012)
	*AtFBN3b*				
Group 4	*AtFBN4*	*Arabidopsis thaliana*	Plastoglobules	Played a role in PQ-9 transportation between plastoglobules and thylakoids. Improved plant immunity.	(Jones et al., 2006; Singh et al., 2010; Lundquist et al., 2012)
	*SlFBN4*	*Solanum lycopersicum*	Plastoglobules	Might be involved in controlling tomato fruit ripening and response to pathogen infections.	(Sun et al., 2022)
	*MdFBI4b*	*Malus domestica*	Plastoglobules	Played a role in response to pathogen infections.	(Singh et al., 2010)
Group 5	*AtFBN5*	*Arabidopsis thaliana*	Stroma	Played a role in PQ-9 synthesis.	(Lundquist et al., 2012; Kim et al., 2015; Otsubo et al., 2018)
	*SlFBN5*	*Solanum lycopersicum*	Stroma	Regulated the expression of JA production under high-light stress.	(Otsubo et al., 2018)
Group 6	*AtFBN6*	*Arabidopsis thaliana*	Thylakoids	Played a role in ROS homeostasis and sulfate metabolism.	(Lundquist et al., 2012; Lee et al., 2020)
	*CsaFBN6*	*Cucumis sativus*	Thylakoids	Upregulated expression under high-light and low-temperature stress.	(Kim et al., 2018)
	*SlFBN6*	*Solanum lycopersicum*	Thylakoids	Engaged in ABA response pathway directly or indirectly.	(Sun et al., 2022)
Group 7	*AtFBN7a*	*Arabidopsis thaliana*	Plastoglobules	ND	(Lundquist et al., 2012)
	*AtFBN7b*				
	*GmFBN7a*	*Glycine max*	Plastoglobules	They strongly upregulated under flood stress.	(Mutava et al., 2015)
	*GmFBN7b*				
	*SlFBN7a*	*Solanum lycopersicum*	Plastoglobules	May have a role in the early differentiating of tomato fruits.	(Sun et al., 2022)
	*SlFBN7b*				
Group 8	*AtFBN8*	*Arabidopsis thaliana*	Plastoglobules	ND	(Lundquist et al., 2012)
	*SlFBN8*	*Solanum lycopersicum*	Plastoglobules	Increased with fruit maturity.	(Sun et al., 2022)
Group 9	*AtFBN9*	*Arabidopsis thaliana*	Thylakoids	ND	(Lundquist et al., 2012)
	*SlFBN9*	*Solanum lycopersicum*	Thylakoids	Increased with fruit maturity.	(Sun et al., 2022)
Group 10	*AtFBN10*	*Arabidopsis thaliana*	Plastoglobules and thylakoids	ND	(Lundquist et al., 2012)
Group 11	*AtFBN11*	*Arabidopsis thaliana*	Thylakoids	Played a role in osmotic stress tolerance.	(Choi et al., 2021)
	*CsaFBN11*	*Cucumis sativus*	Thylakoids	Along with *CsaFBN1* and *CsaFBN6*, it may be implicated in photoprotection to excessive light stress with chilling.	(Kim et al., 2018)
	*SlFBN11*	*Solanum lycopersicum*	Thylakoids	Response to ABA.	(Sun et al., 2022)

FBN, fibrillin; ABA, abscisic acid; JA, jasmonic acid; ROS, reactive oxygen species; ND, not determined.

Gene function modulates gene expression across tissues and developmental stages in different species. FBNs are regulated by various biological and environmental factors during different growth stages (Singh and McNellis, 2011). Research has shown that FBN family members are expressed at different stages of plant development (Pandey et al., 2023). In tomato plants, most of the 14 *SlFBN*s are primarily expressed in the leaves, playing essential roles during leaf development (Sun et al., 2022).

FBN1, a well-conserved FBN found in photosynthetic organisms and terrestrial plants, likely evolved from a precursor, similar to its cyanobacterial counterpart, cFBN1. FBN1 and FBN2, being the most negatively charged proteins in the family, are well-suited for preventing PG coalescence (Ytterberg et al., 2006). FBN1 also plays a role in forming lipoproteic structures in certain chromoplast types, such as PGs or fibrils, contributing to a “sink effect” during pigment accumulation (Simkin et al., 2007). FBN1-suppressed plants exhibit increased susceptibility to infection, suggesting a role in plant defense.

In bell pepper, FBN, homologous to *Arabidopsis* FBN1a, contributes to the reconstitution of fibrils storing polar lipids and carotenoids in the chromoplast, leading to the red coloration of mature fruits due to carotenoid accumulation (Camara et al., 1995). Chloroplast PG levels increased when tobacco overexpression of pepper FBN increases chloroplast PG levels (Rey et al., 2000), indicating that FBN1a and FBN1b regulate PG size by restricting coalescence. These studies collectively demonstrate that clade 1 FBNs are involved in the formation and maintenance of PGs, used by plastids to sequester and store various lipids (Kim and Kim, 2022).

The widespread presence of FBN3 across diverse organisms suggests its critical role in these species. FBN3’s association with modular proteins containing protein-interacting domains (FHA and PB1) (Durocher and Jackson, 2002; Sumimoto et al., 2007) indicates a specialized function, although its exact nature remains unclear without identifying the corresponding interacting proteins.

FBN4, also known as Harpin-binding protein, is localized in chloroplasts, PG, and the PS-II light-harvesting complex in thylakoids (Jones et al., 2006). FBN4 is believed to regulate PG content, influencing photosynthetic activity and stress sensitivity (Flower et al., 2000; Singh et al., 2010). By utilizing stored antioxidants, FBN4 may reduce ROS levels, acting as a mediator of plant stress tolerance (De Pourcq, 2014). FBN4 is also involved in disease, as demonstrated by its role in *Arabidopsis* defense against *Pst* DC3000 (Jones et al., 2006). *FBN4* knockdown in apple and *Arabidopsis* increases susceptibility to bacterial pathogens like Pst DC3000 and *Erwinia amylovora* (Singh et al., 2010). Furthermore, interactions between FBN4 and proteins such as Fd2 or HrpN impact the innate immune response to pathogens.

FBN5, the least conserved FBN in the family, is less understood (Kim et al., 2015). However, the presence of modular FBN5 proteins suggests a potential function in some species. The inclusion of a vesicle transport module (Vps51 and Vps67) in one of these proteins (Zaman and Fancy, 2012) implies a role in vesicle trafficking. FBN5, an *Arabidopsis* stromal protein, binds to solanesyl diphosphate synthases, facilitating PQ-9 production and adaptation to photooxidative stress (Kim et al., 2015). PSII’s electron transport activity is significantly reduced in its absence, while PSI is minimally affected (Kim et al., 2015; Otsubo et al., 2018).

FBN6 is essential for plant response to light stress and ROS scavenging (Lee et al., 2020). It co-immunoprecipitates with phytoene synthase (PSY), the primary enzyme in the carotenoid pathway (Welsch et al., 2018; Iglesias-Sanchez et al., 2023). FBN6 is the only FBN enriched in pure envelope fractions, including PSY (Ferro et al., 2010; Bouchnak et al., 2019).

The absence of FBN7 in algae suggests that its function may be provided by another FBN or is unnecessary in these organisms (De Pourcq, 2014). Localization experiments in *Arabidopsis* indicate that the entire FBN7 sequence, except for a short C-terminal stretch, is required for PG targeting (Vidi et al., 2006; Shanmugabalaji et al., 2013). This targeting is based on proper folding rather than a specific sequence (Vidi et al., 2006; Shanmugabalaji et al., 2013). FBN8 and FBN9 have limited literature, primarily being conserved in terrestrial plants and less in phytoplankton (De Pourcq, 2014). Unlike *FBN2*, *FBN4*, and *FBN6*, *FBN8* and *FBN9* expression increases as tomato fruit matures.

FBN10 is notable for its presence in terrestrial plants, algae, and even distant species like stramenopiles and diatoms (De Pourcq, 2014). It features a dual-module FBN structure, possibly allowing membrane anchoring and fatty-acyl-CoA dehydrogenase activity. However, the function of this domain combination remains unclear. FBN10, like FBN4, may draw diminishing power from PGs (De Pourcq, 2014). FBN10, like FBN4, may derive reducing power from PGs, interacting with fatty acyl-CoA dehydrogenase to provide the necessary reducing power for dehydrogenation/desaturation.

FBN11, the only member of the FBN gene family with a kinase domain between its N-terminus and FBN domain at the C-terminus, suggests a role in signal transduction pathways and regulation (Choi et al., 2021). Its specific presence in terrestrial plants implies a unique regulatory function. *Arabidopsis* FBN11, also known as an OBP3-binding protein, is a putative nuclear-localized protein responsive susceptible to SA. It is hypothesized that FBN11 is restricted to terrestrial plants, possibly involved in root development. During land plant evolution, FBN11 may have reorganized its domains in conjunction with PLAP2 and STT7 kinases, originating from lycophytes (Choi et al., 2021).

## 5 Plant *FBN* genes and abiotic stress

Plants respond to water stress through various mechanisms, including altering growth patterns, increasing antioxidant production, accumulating compatible solutes, and producing stress proteins and chaperones (El-Sappah and Rather, 2022). These responses are mediated by complicated signaling networks, with the *FBN* gene family playing a critical role, particularly in the response to drought and other abiotic stressors (Singh and McNellis, 2011).

Most research has focused on how *Arabidopsis* FBNs function under stress and in response to hormones such as ABA, JA, and salicylic acid (SA) (Verma et al., 2016). These studies aim to understand how FBNs help safeguard plants against environmental stressors. For instance, FBN1a, FBN1b, and FBN2, belonging to groups 1 and 2, have been studied for their role in protecting plants from photodamage caused by PSII (Yang et al., 2006; Youssef et al., 2010). These FBNs regulate JA production for photoprotection, with *FBN1a* showing increased resistance to photoinhibition under light stress when treated with ABA (Yang et al., 2006). Moreover, studies on *abi1* and *abi2* mutants revealed that ABA response regulators, ABI1 and ABI2, alter the expression of *FBN1a* at the transcriptional or post-transcriptional level. ABI2, for example, may regulate protein levels in the cytoplasm before FBN1a translocates to the chloroplast, contributing to the protection against light damage (Yang et al., 2006).

In addition to photoprotection, some FBNs have been implicated in ROS scavenging. For example, FBN6, co-expressed with photosynthetic genes such as *PsbY*, *PsbO*, and *Lhcb6*, is involved in ROS scavenging within the chloroplast thylakoid and envelope membranes under high-light conditions (Lee et al., 2020). *FBN6* may also enhance Cd detoxification and tolerance by improving the ROS scavenging mechanism in chloroplasts. Similarly, *FBN11* expression is induced by abiotic stresses, including ABA, NaCl, and mannitol. Knockout mutants of *FBN11* show decreased seed germination rates in mannitol-containing media, indicating its role in osmotic stress tolerance during seed germination (Choi et al., 2021).

In other crops, drought stress increases the abundance of *CDSP 34* (*FBN1*) mRNA, and protein in potatoes, with immunolocalization studies showing elevated CDSP 34 levels in thylakoids and stroma under drought stress (Eymery and Rey, 1999). In tomatoes, the wild-type *flacca* plant, which accumulates more ABA, showed significantly higher FBN1 protein levels under drought compared to an ABA-deficient mutant (Gillet et al., 1998). *SlFBN11* in tomatoes has been identified as particularly responsive to ABA treatment, indicating its unique role in the ABA signaling pathway (Sun et al., 2022).

In wheat, the expression of *TaFBNA1*, *TaFBNB1*, *TaFBNA2*, *TaFBNB2*, *TaFBND2*, and *TaFBN-B6* is significantly increased under drought, stripe rust, cold, and heat stress (Jiang et al., 2020). Similar upregulation of *FBN1a*, *FBN1b*, and *FBN2* has been observed in the leaves of rice, *Arabidopsis*, *Brassica*, and potato under cold and drought conditions (Gillet et al., 1998; Laizet et al., 2004; Jiang et al., 2020). In chickpeas, *CaFBN1*, *CaFBN2*, and *CaFBN6* expression levels increase under dehydration stress, while *CaFBN3*, *CaFBN5*, *CaFBN7*, *CaFBN8*, *CaFBN9*, *CaFBN10*, *CaFBN11*, and *CaFBN12* show decreased expression under the same conditions (Pandey et al., 2023). The FBN gene family, particularly *GmFBN2*, *GmFBN1*, *GmFBN10*, *GmFBN11*, and *GmFBN15*, has also been implicated in mediating soybean’s response to drought, with significant increases in expression observed (Zafer et al., 2023).

## 6 Conclusion

FBN proteins are a vital family of proteins first identified in the chromoplasts of bell pepper and dog rose. Characterized by a conserved PAP domain, FBNs play essential roles in various plant physiological processes, including photosynthesis, stress responses, and developmental regulation. The FBN family comprises 12 subfamilies, with 11 found in higher plants. Their presence across different plant tissues and developmental stages underscores their versatile functions in growth and environmental response. Notably, FBNs are involved in regulating carotenoid accumulation, chromoplast fibril formation, and protecting against oxidative stress. Their expression is influenced by environmental stressors such as drought, temperature fluctuations, and pathogen attacks, often mediated by plant hormones like ABA and JA. The intricate regulation and numerous activities of FBNs underscore their crucial role in improving plant resilience and adaptation to changing environmental circumstances, particularly abiotic ones.
