# Supplementary material for: Fibrillin gene family and its role in plant growth, development, and abiotic stress
Source: Front Plant Sci. 2024 Oct 29;15:1453974. doi: 10.3389/fpls.2024.1453974 (PMC11580037; doi:10.3389/fpls.2024.1453974)
Supplement: Supplementary file 1 [file DataSheet1.pdf]

Supplementary materials

Fibrillin **(*FBN*) gene family and its role in plant growth, development, and environmental stress**

Ahmed H. El-Sappah, Jia Li, Kuan Yan, ChaoYang Zhu, Qiulan Huang, Yumin Zhu, Yu Chen, Khaled A. El-Tarabily, and Synan F. AbuQamar*

*** Correspondence:**
Synan F. AbuQamar: sabuqamar@uaeu.ac.ae

Supplementary material

Supplementary Figure S1. Distribution of the PAP_fibrillin (PF04755) domain across different taxonomic categories. The presence of the PAP_fibrillin domain is shown in various organisms as identified through InterPro database analysis. The domain is color-coded by taxonomic category: Eukaryota is represented in green, and bacteria is represented in violet. PAP indicates proteins that are typically involved in lipid binding and are associated with plastids, which are key organelles in the cellular context of eukaryotic organisms. The presence and distribution of the PAP_fibrillin domain across these two major taxonomic groups highlight its evolutionary significance and potential functional roles within diverse biological systems. PAP, plastid lipid-associated protein.

Supplementary Table S1. The fibrillin (FBN) families identified in plants.

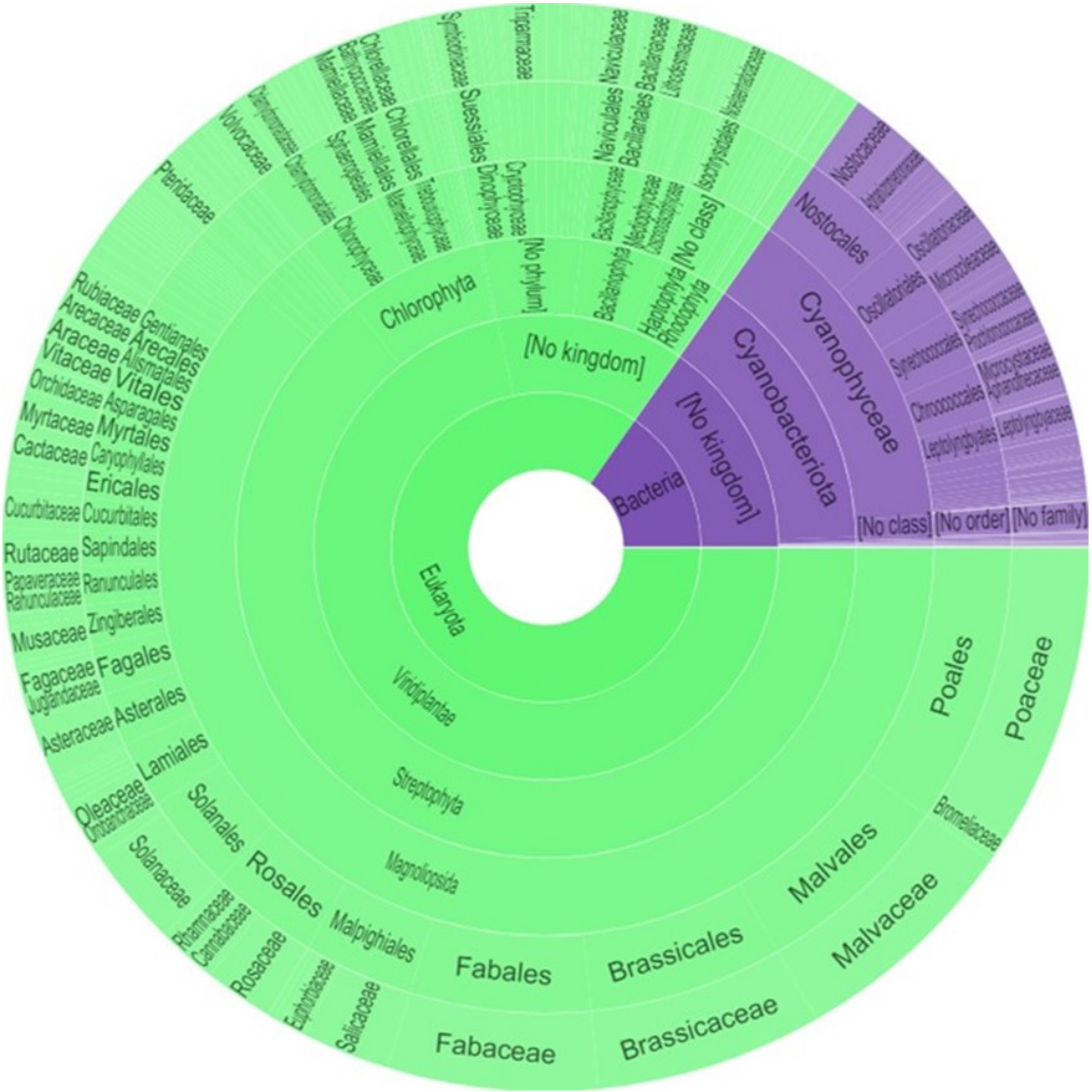

Supplementary Figure S1. Distribution of the PAP_fibrillin (PF04755) domain across different taxonomic categories. The presence of the PAP_fibrillin domain is shown in various organisms as identified through InterPro database analysis. The domain is color-coded by taxonomic category: Eukaryota is represented in green, and bacteria is represented in violet. PAP indicates proteins that are typically involved in lipid binding and are associated with plastids, which are key organelles in the cellular context of eukaryotic organisms. The presence and distribution of the PAP_fibrillin domain across these two major taxonomic groups highlight its evolutionary significance and potential functional roles within diverse biological systems. PAP, plastid lipid-associated protein.

Supplementary Table S1. The fibrillin (FBN) families identified in plants.

Plant	*FBN* gene numbers	References
Arabidopsis thaliana	14	(Singh and McNellis, 2011)
Solanum lycopersicum	14	(Sun et al., 2022)
Cucumis sativus	10	(Kim et al., 2018)
Oryza sativa	11	(Li et al., 2020)
Triticum aestivum	26	(Jiang et al., 2020)
Cicer arietinum	12	(Pandey et al., 2023)
Crocus sativus	10	(Pandey et al., 2023)
Glycine max	16	(Zafer et al., 2023)
Populus trichocarpa	11	(Zafer et al., 2023)
Physcomitrium patens	10	(Zafer et al., 2023)

FBN, fibrillin.

References

Jiang, Y., Hu, H., Ma, Y., and Zhou, J. (2020). Genome-wide identification and characterization of the fibrillin gene family in *Triticum aestivum*. *PeerJ* 8, e9225. doi:10.7717/peerj.9225

Kim, I., Lee, S. C., Kim, E. H., Song, K., Yang, T. J., and Kim, H. U. (2018). Genome-wide identification and expression analyses of the fibrillin family genes suggest their involvement in photoprotection in cucumber. *Plants* 7, 50. doi:10.3390/plants7030050

Li, J., Li, X., Khatab, A. A., and Xie, G. (2020). Phylogeny, structural diversity and genome-wide expression analysis of fibrillin family genes in rice. *Phytochemistry* 175, 112377. doi:10.1016/j.phytochem.2020.112377

Pandey, A., Sharma, P., Mishra, D., Dey, S., Malviya, R., and Gayen, D. (2023). Genome-wide identification of the fibrillin gene family in chickpea (*Cicer arietinum* L.) and its response to drought stress. *Int J. Biol. Macromol*. 234, 123757. doi:10.1016/j.ijbiomac.2023.123757

Singh, D. K., and McNellis, T. W. (2011). Fibrillin protein function: the tip of the iceberg? *Trends Plant Sci*. 16, 432-441. doi:10.1016/j.tplants.2011.03.014

Sun, H., Ren, M., and Zhang, J. (2022). Genome-wide identification and expression analysis of fibrillin (FBN) gene family in tomato (*Solanum lycopersicum* L.). *PeerJ* 10, e13414. doi:10.7717/peerj.13414

Zafer, M. Z., Tahir, M. H. N., Khan, Z., Sajjad, M., Gao, X., Bakhtavar, M. A., Waheed, U., Siddique, M., Geng, Z., and Ur Rehman, S. (2023). Genome-wide characterization and sequence polymorphism analyses of *Glycine max* fibrillin (FBN) revealed its role in response to drought condition. *Genes* 14, 1188. doi:10.3390/genes14061188
